# Worldwide suicide mortality trends by firearm (1990–2019): A joinpoint regression analysis

**DOI:** 10.1371/journal.pone.0267817

**Published:** 2022-05-25

**Authors:** Irena Ilic, Ivana Zivanovic Macuzic, Sanja Kocic, Milena Ilic

**Affiliations:** 1 Faculty of Medicine, University of Belgrade, Belgrade, Serbia; 2 Faculty of Medical Sciences, Department of Anatomy, University of Kragujevac, Kragujevac, Serbia; 3 Faculty of Medical Sciences, Department of Social Medicine, University of Kragujevac, Kragujevac, Serbia; 4 Faculty of Medical Sciences, Department of Epidemiology, University of Kragujevac, Kragujevac, Serbia; Charles Sturt University - Port Macquarie Campus, AUSTRALIA

## Abstract

**Introduction:**

Suicide by firearm is a major public health problem in many countries. But, studies that investigated the mortality of suicide by firearm on a global scale are still limited. The aim of this study was to assess the global, regional and national trends in mortality of suicide by firearm from 1990 to 2019.

**Method:**

Mortality data of suicide by firearm was presented using the age-standardized rates (ASRs, expressed per 100,000). Joinpoint regression analysis was used to assess trends of mortality of suicide by firearm: the average annual percent change (AAPC) with the corresponding 95% confidence interval (95%CI) was calculated.

**Results:**

A total of 52,694 (45,110 male and 7584 female) deaths of suicide by firearm were reported worldwide in 2019. The global ASR of suicide by firearm was six-fold higher in males than in females (1.15 per 100,000 and 0.19 per 100,000, respectively), and varied greatly across countries: the highest rates were in Greenland (24.52 per 100,000 and 2.69 per 100,000, respectively) and the United States of America (10.13 per 100,000 and 1.66 per 100,000, respectively), while the lowest rates (0.05 per 100,000 or less) were observed in China, Japan and Singapore. Globally, the mortality of suicide by firearm had a decreasing tendency from 1990 to 2019 in both sexes together (AAPC = -2.0% per year; 95%CI = -2.1 to -1.9).

**Conclusion:**

Decreasing trends in mortality of suicide by firearm were observed in majority of countries across the world, but not in all. Future research should determine more effective ways to further reduce mortality of suicide by firearm.

## Introduction

Suicides by firearm are one of major public health issues worldwide [1–5]. Based on the World Health Organization (WHO) 2019 estimates, suicides by firearm caused more than 50,000 deaths in the world in 2019 [1]. The correspondence analysis of the WHO mortality database showed opposite international patterns in preferred suicide methods, and indicated that firearm and pesticide suicides tend to substitute traditional methods, such as hanging, in many countries [6].

In 2016, firearm suicides in the United States of America represented 35.3% of global firearm suicides [2]. Among central European countries, Switzerland has a high rate of suicide by shooting, particularly in men [6,7]. Firearm suicides were widely prevalent in men in Croatia and Serbia, following the civil wars in the 1990s [6,8]. Firearms for suicide were mainly used by men, especially army weapons [2]. International studies show a positive correlation between the rates of suicide by firearm and the availability of and attitudes towards guns [6,9–12].

In Norway, over the 1969–2012 period, firearm suicide rates in males reached a maximum in 1988 (at about 12 per100,000) with an annual change of 4.6%, and then decreased through 2012 (at about 4 per 100,000) with an annual change of −4.8%; in contrast to younger age groups, the increase in the rates of suicides by firearms was observed in the 65 years and over only [13]. The firearm suicide mortality from 1981 to 2018 in Canada had been significantly declining in both sexes, with no significant changes recorded only in recent years among both sexes and across almost all age groups, except the oldest age group (65+ years) [14]. While the trend in firearm suicide in the United States of America was decreasing from 1999 to 2006 by -1.1% per year, from 2006 onwards it showed a significant increase in death rates of +2.0% per year [15]. Additionally, among those aged 5 to 24 years in the United States of America, after a statistically significant decline in suicide mortality from firearms from 1999 to 2007 (by -3.83% per year), subsequently a statistically significant increase in suicide by firearms was noted to 2018 [16]. Changes in the rates and trends of suicide by firearm have been attributed, partly, to adoption of firearms legislation [17,18]. A recent comprehensive review of firearm-control legislation worldwide identified the association between firearm-related laws (regarding restrictions on purchase, access, or use of firearms) and suicide deaths by firearm in certain countries [17]. After the firearm legislation reform in the European Union in 1997, Kapusta and coauthors [19] found that the law was associated with reductions in firearm suicide in Austria (change in trends in pre-/post-law periods = −9.9%). Similarly, Australia’s 1996 gun law reforms were followed by more accelerated declines in suicide mortality by firearm, with the ratio between pre-and post-law trends of 0.954 [20].

Lower socioeconomic status is associated with suicide [21], but this finding was not consistent [22]. According to the Australian national data, there was a positive correlation between suicide rates and Human Development Index values, as a marker of well-being [21]. Naghavi and coauthors found that the Socio-Demographic Index (as a composite measure of income per capita, fertility, and education level) was not associated with firearm suicides [2]. However, research is still lacking to clarify ambiguities regarding the relationships between suicides by firearm and the well-being of a population at the national and global levels.

WHO and the United Nations Sustainable Development Goals (UNSDGs), published in 2015, target to reduce premature mortality from non-communicable diseases by one third by 2030, through prevention and treatment, and promote mental health and well-being [23]. Nevertheless, studies that investigated mortality of suicide by firearm on a global scale are still limited. A better understanding of trends in suicide by firearm can help health service providers and policy makers to identify more effective public health strategies for reducing suicide mortality. The objective of this manuscript was to assess global, regional and national trends in mortality of suicide by firearm. Additionally, this study aimed to evaluate the possible association between mortality of suicide by firearm and some selected measures of human development (Gross Domestic Product—GDP, Gross Domestic Product per capita—GDP per capita, the Human Development Index—HDI).

## Methods

### Study design

For this descriptive epidemiological study, the annual underlying cause of death data was used to describe trends in mortality from suicide by firearm for the period 1990–2019.

### Data source

Mortality data of suicide by firearm were extracted from the Global Burden of Disease (GBD) Study [24]. For self-harm by firearm, based on the 10th revision of the International Classification of Diseases [25], the GBD cause list covered codes X72-X74.9, including: site code X72 (Intentional self-harm by handgun discharge), X73 (Intentional self-harm by rifle, shotgun, and larger firearm discharge), and X74 (Intentional self-harm by other and unspecified firearm discharge).

The GBD database provides a comprehensive and comparable assessment for mortality of suicide by firearm across the world [24]. The GBD estimates are based on multiple relevant data sources of varying completeness and quality, incorporated using consistent methods for data standardization and adjustments for incomplete data. According to the WHO guidelines, the definition of the underlying cause of death includes a disease or injury that has started a series of diseases or an injury that has triggered a series of disease states that directly led to death [25]. For countries without high-quality vital statistics (either due to under-reporting of deaths, unknown age and sex, or high percentage of ill-defined cause of death) adjustment of mortality data was computed using standard methods (for example using other data, including household surveys, verbal autopsy, sample or sentinel registration systems, special studies, etc.) [24]. The GBD estimates have been developed and documented following the Guidelines for Accurate and Transparent Health Estimates Reporting (GATHER) [26].

We extracted data for suicide in men and women for 204 countries and territories worldwide, for every year from 1990 to 2019 [24]. Mortality of suicide by firearm was presented within six WHO regions: Africa, Americas, South-East Asia, Europe, Eastern Mediterranean, and Western Pacific. For this objective, the age-standardized mortality rates (ASRs, expressed per 100,000 persons) by using the time-invariant world standard population developed for the GBD Study, were extracted. Specific (by age and sex) mortality rates (expressed per 100,000 persons) were presented. The subgroup analyses were performed: the age groups were divided in four strata (10–24 / 25–49 / 50–69 / 70+ years), for males and females separately. As there were very few suicides by firearm deaths reported among those under the age of 10 in most populations and due to difficulty in determining intent in children, the results are not shown for ages <10 years.

Data for the GDP (in US dollars), GDP per capita and HDI were obtained from the United Nations data for National Accounts Main Aggregates database [27]. The Human Development Index (HDI) is a summary measure of average achievement in key dimensions of human development: the health dimension (assessed by life expectancy at birth), the education dimension (assessed by mean of years of schooling for adults aged 25 years and more and expected years of schooling for children of school entering age) and the standard of living dimension (assessed by gross national income per capita). The value of the HDI ranges from 0 to 1, where 1 indicates the highest, ideal development.

### Statistical analysis

The magnitude and direction of temporal trends for mortality of suicide by firearm were assessed using the joinpoint regression analysis (Joinpoint regression software, Version 4.8.0.1 –April 2020, available through the Surveillance Research Program of the US National Cancer Institute), proposed by Kim et al [28]. The joinpoint regression analysis is based on the algorithm that tests whether the trends in the annual age-standardized mortality rates of suicide by firearm fit to a series of joined straight lines on a log scale. Line segments are joined at points called joinpoints, which denote a statistically significant change in trend and determine the trends between joinpoints. The permutation test is applied to identify the number of significant joinpoints, and each permutation test is estimated using Monte Carlo method. For this study, the analysis starts with a minimum of zero joinpoints (i.e. a straight line) and tests whether a change in the trend was statistically significant by testing more joinpoints up to the maximum of four joinpoints (five line segments) which were allowed for each model. The grid search was used to fit the segmented regression function [29]. Using the calendar year as a regression variable, the joinpoint regression analysis estimated the annual percentage change (APC) in rates between change points, with its 95% confidence intervals (95%CI). The average annual percent change (AAPC) as a summary measure of the trend over a pre-specified fixed interval, with the corresponding 95% confidence interval (95%CI) was determined [30]. By contrast to global and regional level, in order to more clearly present the results in this manuscript, the mortality trends of suicide by firearm by country across the world were presented with a straight line in the whole period, even if there were changes in trends in the observed period.

For describing the direction of temporal trends, the terms “significant increase” or “significant decrease” were used, in order to signify that the slope of the trend was statistically significant (p<0.05, on the basis of the statistical significance of the AAPC compared to zero). For non-statistically significant trends (p>0.05, while AAPC with a 95% CI overlapping with zero), the terms “non-statistically significant increase” (for AAPC >0.5%), and “non-statistically significant decrease” (for AAPC < -0.5%) were used, while the term “stable” was used for AAPC between -0.5% and 0.5%. Disparities in trends in mortality of suicide by firearm according to sex and age were tested by using a comparability test [31]. The purpose of the comparability test was to compare two joinpoint regression lines and determine whether the two segmented line regression functions are identical (test of coincidence) or parallel (test of parallelism). A p value <0.05 was considered statistically significant for all tests.

The association between mortality of suicide by firearm and measures of human development (GDP, GDP per capita, and HDI) was estimated with a simple regression model. The correlation between mortality of suicide by firearm and measures of human development (GDP, GDP per capita, and HDI) was estimated with a Pearson Correlation coefficient. Statistical significance was accepted at the level of p<0.05; statistical analyses were conducted using the SPSS Software (version 20.0, Chicago, IL).

### Ethics statement

This study was approved by the Ethics Committee of the Faculty of Medical Sciences, University of Kragujevac (No. 01–14321). The study was conducted using publicly available data. The data are derived from the official databases and are fully aggregated, without any identification data, and no patient approvals were required for the design, or conduct, or reporting or dissemination of the study.

## Results

**Fig 1:**
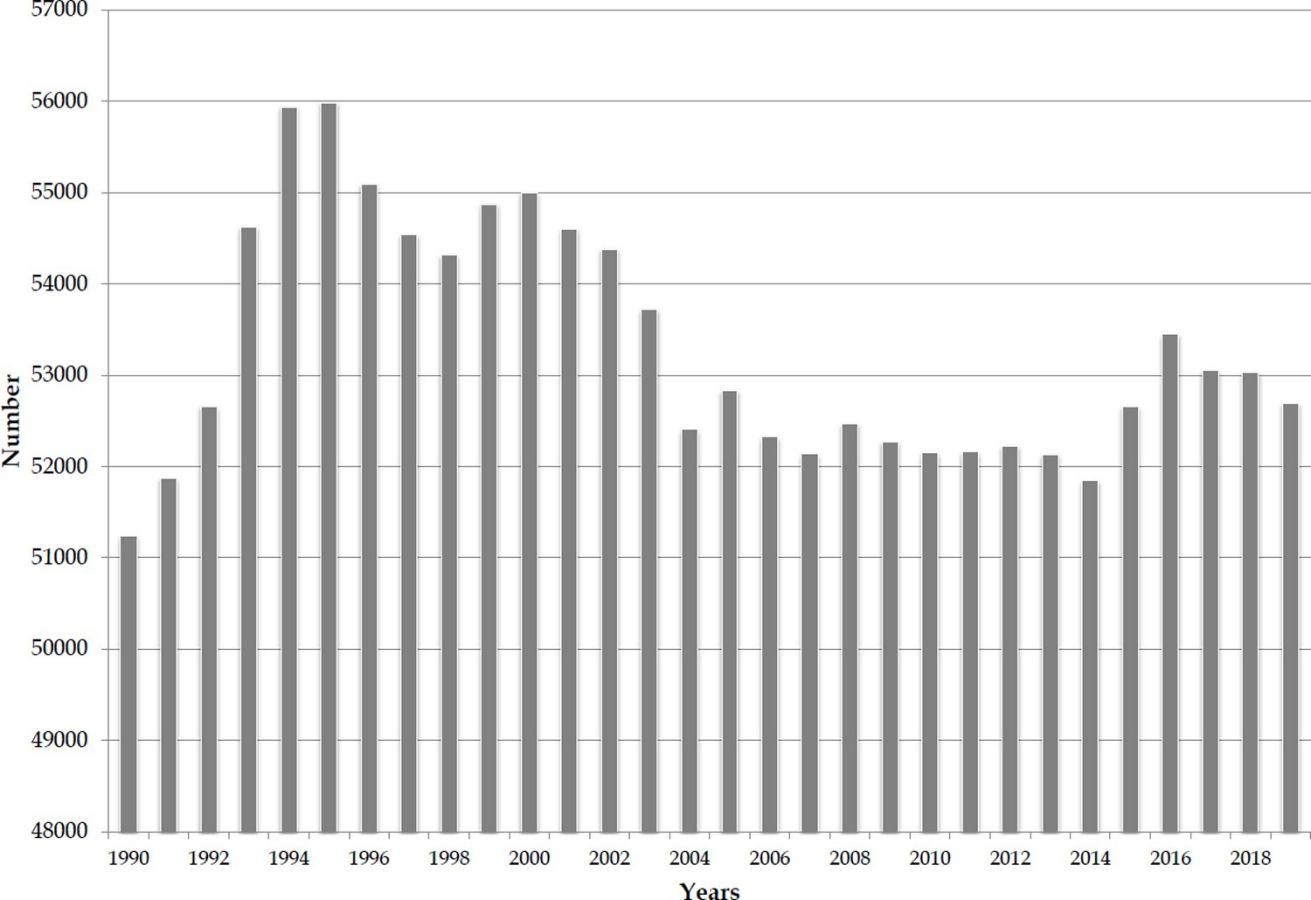
Global suicide deaths by firearm, 1990–2019. Source: GBD estimates [20].

A total of 1.6 million suicide deaths by firearm occurred worldwide during the observed period (1.4 million among males and 0.2 million among females) (Fig 1). Annually, the number of suicide cases by firearm ranged from 51,241 in 1990 to 55,983 in 1995. A total of 52,694 (45,110 male and 7584 female) suicide deaths resulting from firearms were reported worldwide in 2019.

**Fig 2:**
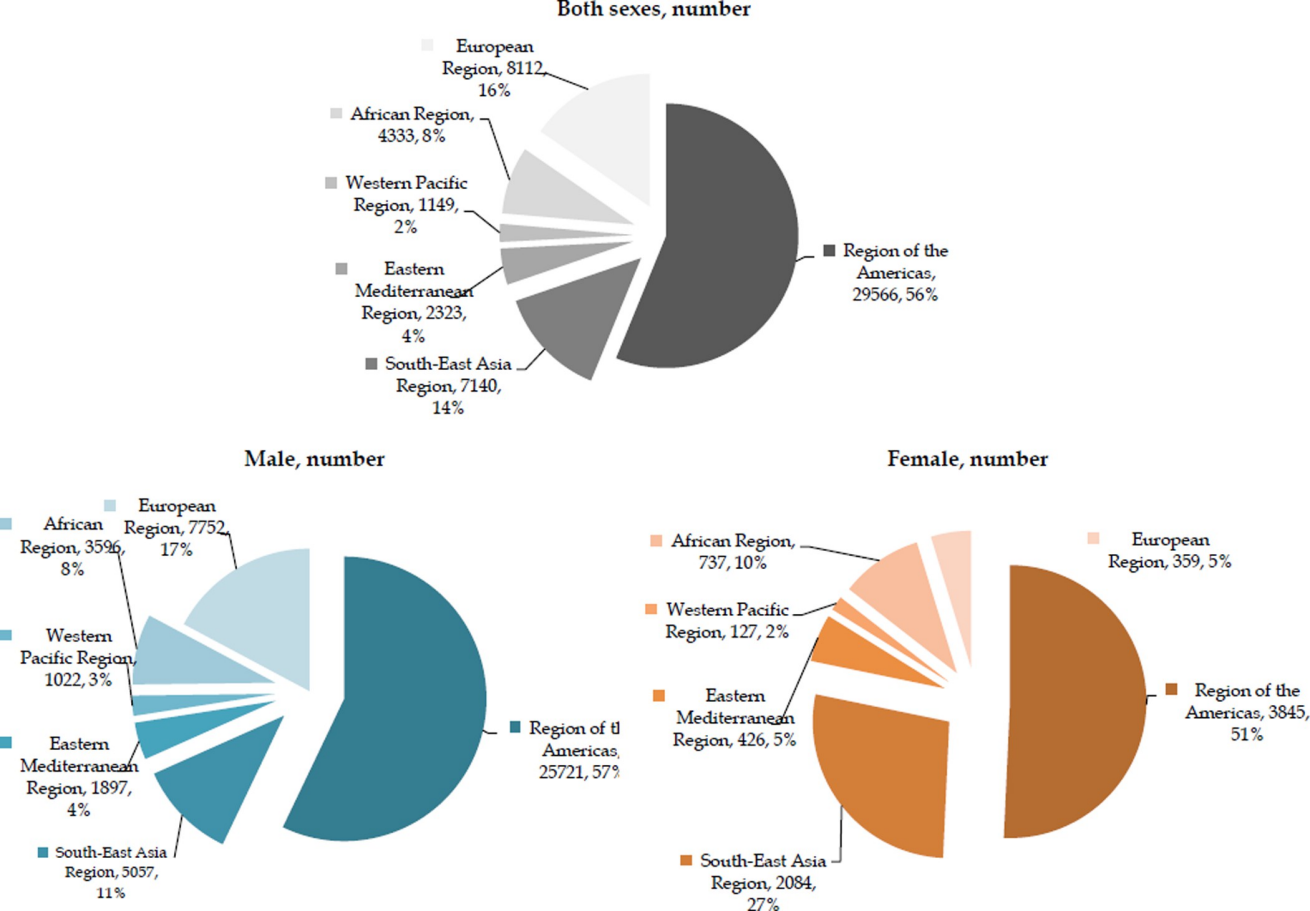
Number of suicides by firearm (global and by WHO regions), by sexes, 2019. Source: GBD estimates [20].

In both sexes, most suicide deaths by firearm (29,566; 56% of the total) were recorded in the American region, and then in the European Region (8,112; 15%) and South-East Asia Region (7,140; 15%) (Fig 2). The distribution of suicides resulting from firearm are similar for both males and the general population. In females, the highest percentage of suicides by firearm is evident in the American region (3845; 51% of the total), followed by the South-East Asia Region (2084; 27%). Percentage of suicides by firearm among females in the Europe region was three times as low (5%) in comparison to males (17%), while in the South-Asia region it was three times as high (27% vs. 11%, respectively).

**Fig 3:**
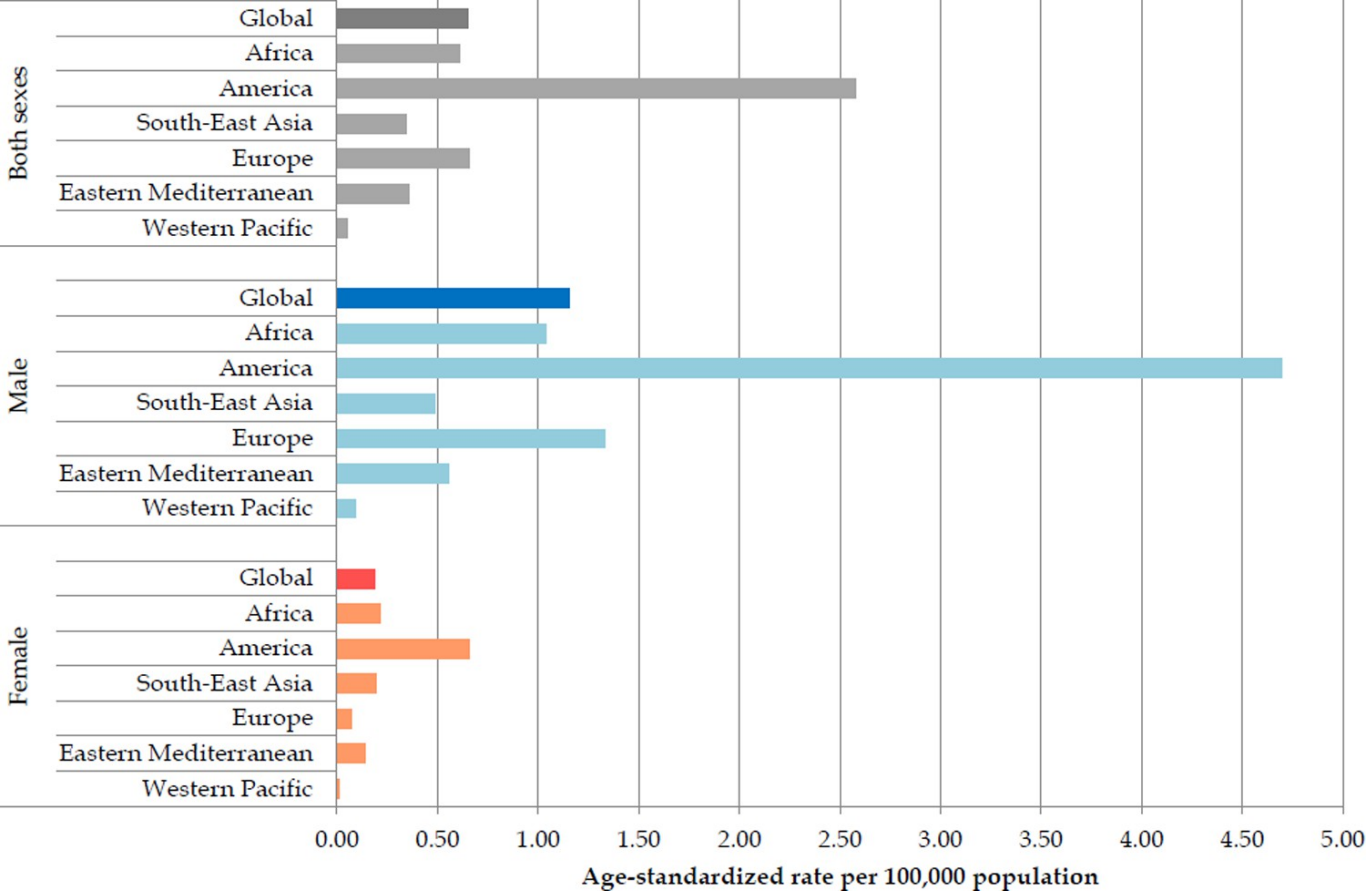
Age-standardized mortality rates (global and by WHO regions) of suicide by firearm, by sexes, 2019. Source: GBD estimates [20].

The global age-standardized rate of mortality of suicide by firearm was 0.65 per 100,000 persons (Fig 3). The highest rate was found in the American region (2.58 per 100,000), while the lowest rate was reported in the Western Pacific Region (0.05 per 100,000). The global ASR of suicide mortality in 2019 was six-fold higher in males than in females (1.15 per 100,000 in males vs. 0.19 per 100,000 in females). Both in males and females, the mortality of suicide by firearm was the highest in the American region (4.69 per 100,000 vs. 0.66 per 100,000, respectively). Both in males and females, the lowest suicide mortality rates in 2019 were observed in the Western Pacific Region (0.09 per 100,000 and 0.01 per 100,000, respectively).

**Fig 4:**
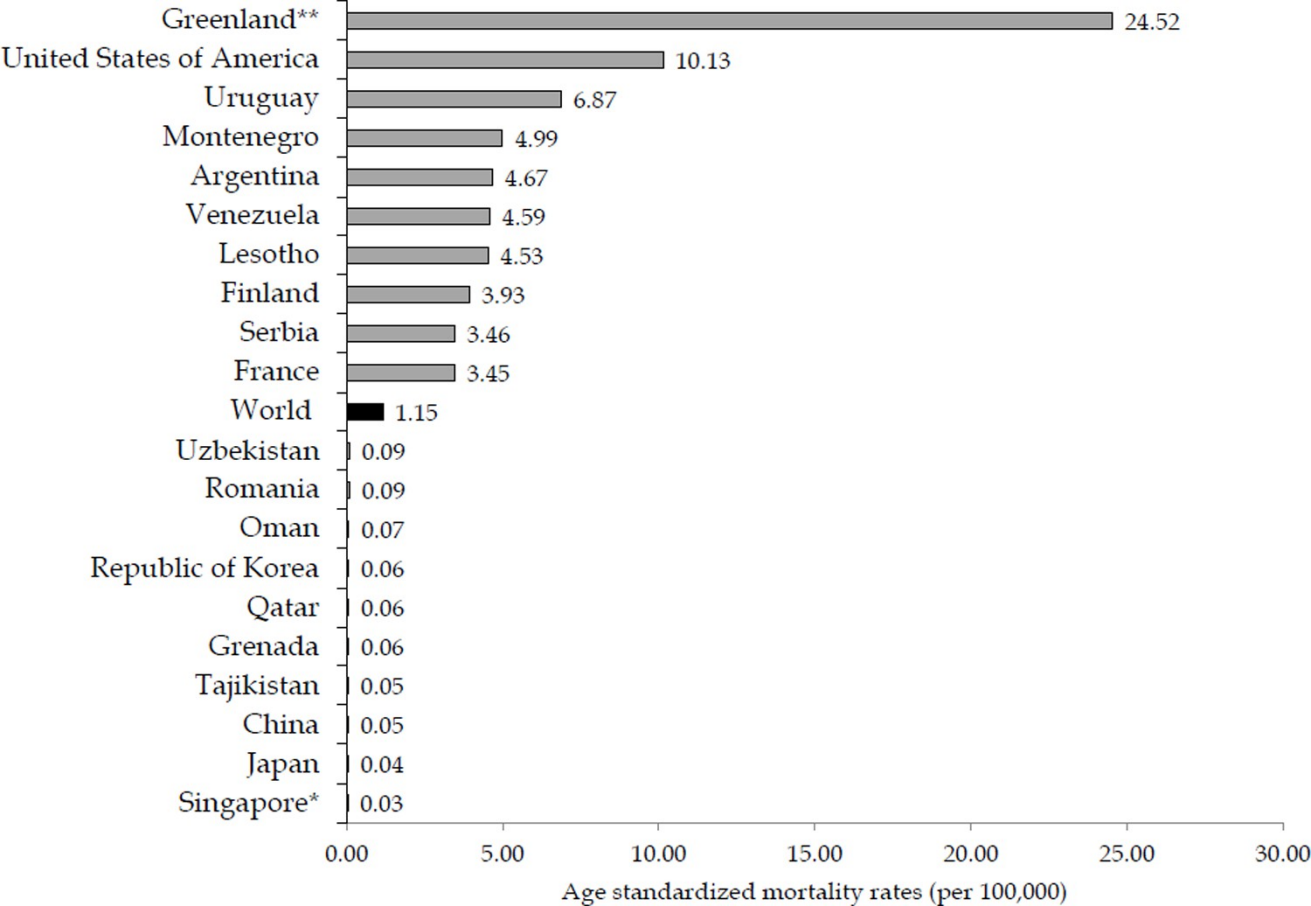
Suicide mortality by firearm in men, by countries: 10 highest / 10 lowest rates in the world in 2019. * Country with the lowest rate; ** Country with the highest rate. Source: GBD estimates [20].

**Fig 5:**
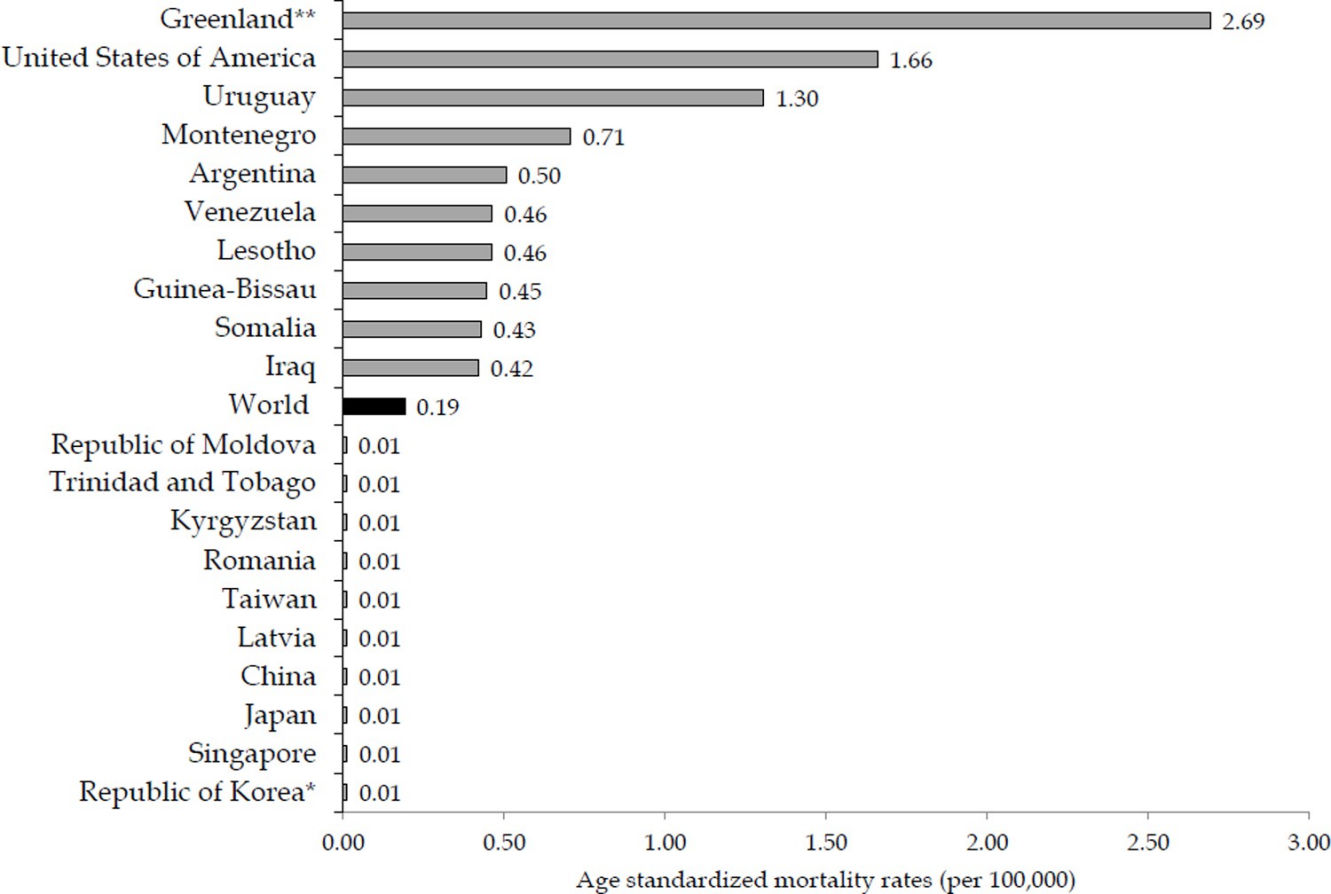
Suicide mortality by firearm in women, by countries: 10 highest/10 lowest rates in the world in 2019. * Country with the lowest rate; ** Country with the highest rate. Source: GBD estimates [20].

There are significant variations in the mortality of suicide by firearm between countries by sexes in 2019 (Figs 4 and 5). Mortality rate of suicide in males was the highest in Greenland (24.52 per 100,000), and then the United States of America (10.13 per 100,000) and Uruguay (6.87 per 100,000), followed by populations in Montenegro, Argentina, Venezuela and Lesotho (equally about 5 per 100,000 people), while the lowest mortality rates (0.05 or less per 100,000 people) were observed in Tajikistan, China, Japan and Singapore (Fig 4). The mortality of suicide in females varies greatly across countries: mortality rate was the highest in Greenland (2.69 per 100,000), followed by the United States of America (1.66 per 100,000) and Uruguay (1.30 per 100,000), while the lowest mortality rate (equally 0.01 per 100,000 people) was observed in Republic of Korea, Singapore, Japan, China (Fig 5).

In comparison to males, mortality rates of suicide by firearm were lower in females in all countries across the world in 2019 (S1 Table). Globally, from 1990 to 2019, age-standardized mortality rates of suicide by firearm have a decreasing tendency in both sexes together (AAPC = -2.0% per year). Both in males and females, suicide mortality rates by firearm decreased by AAPC = -2.1%, and by AAPC = -1.6%, respectively. According to the comparability test, suicide mortality trends in males and females were not parallel and not coincident (p<0.05). When mortality trend of suicide by firearm was analyzed by six WHO regions, significantly decreasing trends were observed in males in five regions: in Western Pacific (AAPC = -3.8%), Europe (-3.2%), South-East Asia (-1.9%), Americas (-1.5%), and Eastern Mediterranean (-0.5%); the only exception was the region of Africa, with a stable trend of suicide mortality (+0.3%). Similarly, significantly decreasing trends were observed in females in five regions: in Western Pacific (AAPC = -4.6%), Europe (-3.3%), South-East Asia (-2.3%), Africa (-1.9%), and Americas (-1.3%); the only exception was the Eastern Mediterranean region, with a stable trend of suicide mortality (+0.0%). In both sexes together, 150 out of the total 204 countries and territories across the world showed a significantly decreasing trend in mortality of suicide. Out of all 150 countries with a decline in suicide mortality, Singapore (AAPC = -6.8%), Sri Lanka (AAPC = -5.9%), Australia (AAPC = -5.6%), and Switzerland (AAPC = -5.5%) showed the most marked reductions in mortality rates. A total of 31 countries had a significant increase in suicide mortality, with Jamaica (AAPC = +11.5%) and Lesotho (AAPC = +4.4%) showing the highest increase in mortality. In several countries, suicide mortality trends were significantly increasing in both males and females (in Armenia, Egypt, Eswatini, Jamaica, Lesotho, Libya and Zimbabwe), with the magnitude of the increase mostly higher in females. In some countries a significant increase of trends in suicide mortality was seen only in women, but not in men (in Afghanistan, Antigua, Azerbaijan, Belize, Botswana, Cuba, Lebanon, Mauritius, Morocco, Sudan, Tunisia and Yemen).

Suicide death rates by firearm decreased with age both in males and females (Table 1). In males, suicide mortality rates were almost two times higher in people aged 70 or older than in people under 70, while in females this difference was not observed. Age-specific death rates of suicide by firearm in males were higher than in females in all age groups: difference was about three times in the youngest age group of 10–24 years, while a fifteen-fold higher rate was observed in males than in females in the oldest age group of 70+ years. Suicide mortality by firearm was significantly decreasing in all age groups in both sexes from 1990 to 2019. According to the comparability test, suicide mortality trends by age were not parallel (p<0.05) neither in males or in females.

**Table 1:** Joinpoint regression analysis: Global trends* in age-specific mortality rates (per 100,000) of suicide by firearm, by sex, 1990–2019.

		Males			Females		
Age**		*Age-specific rates*		AAPC (95% CI)	*Age-specific rates*		AAPC (95% CI)
		1990	2019		1990	2019	
10–19		0.62	0.35	- 2.4* (-2.6 to -2.1)	0.22	0.15	- 1.7* (-1.9 to -1.5)
20–29		2.01	1.14	- 2.3* (-2.4 to -2.2)	0.45	0.27	- 2.1* (-2.2 to -1.9)
30–39		2.22	1.18	- 2.5* (-2.6 to -2.3)	0.43	0.24	- 2.1* (-2.3 to -1.9)
40–49		2.50	1.45	- 2.3* (-2.6 to -2.1)	0.42	0.27	- 1.7* (-1.8 to -1.6)
50–59		2.75	1.96	- 1.1* (-1.3 to -0.9)	0.33	0.26	- 0.4* (-0.6 to -0.3)
60–69		3.65	2.35	- 1.5* (-1.6 to -1.3)	0.34	0.24	- 0.9* (-1.2 to -0.5)
70–79		6.19	3.24	- 2.6* (-2.9 to -2.4)	0.39	0.24	- 2.1* (-2.4 to -1.8)
80–89		10.70	5.65	- 2.4* (-2.5 to -2.3)	0.41	0.26	- 1.8* (-2.0 to -1.7)
90+		11.94	8.25	- 1.4* (-1.6 to -1.2)	0.57	0.41	- 1.3* (-1.4 to -1.2)

* Statistically significant trend (p<0.05)

** Joinpoint results are not shown for the subgroups aged <10 years for mortality, because fewer than 5 cases of suicide cases occurred in each of the decennium in any year. AAPC, for full period presented AAPC (Average Annual Percent Change); CI = Confidence Interval.

The linear regression showed significant negative association of global suicide mortality of firearm with HDI, GDP and GDP per capita both in males (R^2^ = 0.984, p<0.001; R^2^ = 0.935, p<0.001; R^2^ = 0.928, p<0.001, respectively) and females (R^2^ = 0.973, p<0.001; R^2^ = 0.906, p<0.001; R^2^ = 0.906, p<0.001, respectively) (S1 Fig).

The Pearson coefficient showed significant positive correlation between mortality of suicide by firearm in males and GDP (r = 0.370) and GDP per capita (r = 0.201) by countries in 2019 (equally both at p<0.01), and absence of correlation with HDI (r = 0.088, p>0.05) (S2 Fig). The significant positive correlation was reported between mortality of suicide by firearm in females and GDP by countries in 2019 (r = 0.403, p<0.01), with absence of correlation with GDP per capita (r = -0.047, p>0.05), while significant negative correlation was noted between mortality of suicide by firearm in females and HDI (r = -0.257, p<0.01).

## Discussion

The present study describes global, regional and national trends of mortality from suicides by firearm across the world during the last three decades. Despite the decreasing trends in mortality of suicide by firearm in most of the areas, observed in both sexes and in all age groups, in 31 countries increasing trends in mortality of suicide by firearm were reported.

In 2019, an estimated total of 52,694 suicide deaths resulting from firearms occurred in the world, with the ASR of 0.65 per 100,000 people in both sexes together. Throughout the whole observed period, suicide mortality rates by firearm were the highest in developed countries (such as the United States of America, France, Canada, Finland, Switzerland, Norway), despite a significant declining trend. At the same time, firearm suicide death rates are over five times higher in the United States of America than they are in other high-income countries. On the other hand, in developing countries (such as Venezuela, Lesotho, Central African Republic, Eswatini, Mozambique) where low mortality rates from suicide by firearm were recorded at the beginning of the observed period, significantly higher rates were recorded in 2019 for both sexes. Marked geographic variations in mortality rates of suicide by firearm could be explained by different prevalence of the main risk factors (such as some socio-demographic characteristics, firearms ownership and availability, large alcohol consumption, comorbidity, etc), and variations in suicide prevention [2,4,32,33]. Besides, it is always a question whether the differences in mortality of suicide by firearm are real or partialy reflect variations in data quality worldwide, in the process of registering causes of death or under-reporting [34,35].

The available literature on the association between firearm-related laws and the rate of firearm-related suicides has reported on studies regarding the legislations and rate of suicide by firearm in high income countries [17–20], mainly in the United States of America [17,18,36]. A longitudinal study of US states from 2012 to 2016, indicated that the total number of firearm laws and Gun Violence Restricting Order laws was negatively associated with firearm-related suicide rate among older adults ages 55–64 and >65 years-old (p<0.001) [36]. In New Zealand, a study which examined national suicide data for 8 years before, and 10 years following the introduction of a more restrictive firearms legislation (Amendment to the Arms Act, 1992) showed a significant decrease in rates of firearm-related suicides, particularly among youth [37]. However, a cross-sectional state-level study in the United States of America showed that not all of the firearm laws were associated with reduced mortality of suicide by firearm: whiles reductions in firearm-related suicide deaths were associated with law that enforced firearm identification and permit processes, the increased suicide rates were associated with three laws and the remaining 20 laws were inconclusively associated [38]. Apart from the variety of laws / the differences in law implementation methods / the diversity in law enforcement efforts / the severity of punishments associated with legal violations / specifics of firearm-related policies across countries, some confounding social and state-level factors act both before and after the respective laws [19,38]. For example, some of the countries with the highest mortality rates of suicide by firearm have the highest social well-being indicators (GDP, GDP per capita, HDI) at the same time (the United States of America, Norway, France, Finland, Canada, Denmark). Also, our study showed a positive correlation between mortality of suicide by firearm in both sexes and GDP in 204 countries and territories worldwide in 2019. In contrast, our study reported a significantly negative correlation only between mortality of suicide by firearms in women and HDI by countries in 2019, which should be evaluate in future epidemiological analytical research.

In this study, men had higher rates of suicide by firearm in all countries, and in all age groups. Epidemiological studies suggested an association between mortality of suicide by firearm and certain socio-demographic characteristics (male sex, middle-age, occupation that provides ready access to firearms such as farmers, farm workers, forestry workers and veterinary surgeons, or active service or retired from the armed services, or the security forces), large amount of alcohol consumption, a recent physical illness, marital problems or a breakdown in relationships [2,39–41]. By contrast, contact with mental health services in the year prior to their death, diagnosis of depression or severe mental illness was not a feature of firearm suicides (this may reflect sex differences in help-seeking) [32,40].

Globally, a substantial decrease in mortality trends of suicide by firearm was observed both in males and females. The decrease in mortality rates of suicide by firearm can primarily be attributed to the decrease in rates in the Americas and European regions (from 7.15 per 100,000 and 2.73 per 100,000 persons in 1990 to 4.69 per 100,000 and 1.33 per 100,000 in 2019 in males, and from 0.99 per 100,000 and 0.16 per 100,000 in 1990 to 0.66 per 100,000 and 0.07 per 100,000 in 2019 in females). But, a total of 31 countries had an increase in mortality of suicide by firearm, although they were mostly less developed countries. Although reasons are not yet well understood, implementation of national guidelines for the suicide prevention only in some countries might, at least partly, explain the observed international differences in mortality rates and trends of suicide by firearm [42]. Numerous studies suggest that restrictive firearm legislation should be an imperative part of a country’s suicide prevention programme [7,9]. The introduction of specific prevention programmes focusing on firearms, such as firearm legislation reform, led to a decrease in suicide rate in the United States of America [15,43], Austria [12], Australia [20]. But, some studies showed opposite findings [17,44]. Some studies, that analized the impact of the Australian Gun Buyback program and the Australian National Firearms Agreement on suicide mortality rates in the Australian population, showed that there was no statistically observable additional impact on suicide mortality attributable to firearms [45,46]. Consequently, although most researchers agree that there is evidence for the effectiveness of restricting access to firearm in prevention of suicide, the above studies suggest that a much more dimensional strategy to reduce firearm-related suicide deaths is needed. A recent study in the United States of America found an association between firearm-related mortality rates among youth and county-level poverty [47]. Some other studies showed that firearms suicide rates were associated with rates of unemployment, low income rates, consumption of alcohol per capita [2,48–50]. Our study showed significant negative association of global trends in suicide mortality of firearm with HDI, GDP and GDP per capita both in males and females. Similarly, findings of other studies confirmed significant negative correlation between economic factors (such as the HDI, GDP per capita, GINI index) and suicide rates in both genders [51–53].

Therefore, the differences between the regional and national rates and trends in mortality of suicide by firearm indicate further possibilities for reducing suicide mortality and the need for a more effective public health approach to prevention of suicide across the world.

### Strengths and limitations

This study presented comprehensive global, regional and national trends in mortality of suicide by firearm in the last three decades. Additionally, the joinpoint regression analysis provides estimation of magnitude and direction of trends in mortality of suicide by firearm. The presented trends are crucial not only for monitoring and assessment of the epidemiological features of the suicides in the world, but also for evaluation of preventive measures. The international differences in suicides mortality rates and trends emphasize the need for a more effective public health approach to prevention of suicide worldwide. Finally, this study represents a substantial improvement from some previous studies, with less countries comprised, a shorter time period observed, with a linear trend assessment only.

But, several sources of limitations in this study should be taken into account. Firstly, the accuracy of the GBD estimates generally depends on the availability of data, that can potentially lead to inherent bias from incomplete data sampling in some locations and standardization techniques used. Further, a limitation specific to evaluating firearm-related deaths can lead to under-estimation or over-estimation in mortality of suicide by firearm: namely, misclassification of cause of death, either unintentional firearm injury deaths were misclassified as suicide or homicide, or intentional firearm deaths were wrongly classified as unintentional deaths, can be linked to certain cultural or religious circumstances in some regions. Despite these shortcomings, this study provides useful insights into the variations in the mortality of suicide by firearm worldwide and could provide help for health authorities and policy makers to develop more effective suicide prevention strategies based on reliable estimates of the mortality of suicide by firearm.

## Conclusion

There are large international differences in the mortality patterns of suicide by firearm. Despite the decreasing trends recorded in both sexes in most countries in the world from 1990 to 2019, the suicide by firearm showed an increasing trend in some countries. Future research should investigate the factors which have contributed to substantial geographic variations in mortality of suicide by firearm in order to determine more effective ways to further reduce mortality in suicide by firearm.

## Supporting information

S1 Fig Association of global mortality of suicide by firearm (age-standardized rate, ASR) with Gross Domestic Product (GDP), GDP per capita and Human Development Index (HDI), by sexes, 1990–2019. (TIF)

S2 Fig Correlation of mortality of suicide by firearm (age-standardized rate, ASR) with Gross Domestic Product (GDP), GDP per capita and Human Development Index (HDI), by countries and sexes, in 2019. (TIF)

S1 Table Mortality of suicide by firearm, by location and sex, 1990–2019; a joinpoint analysis. (DOC)
