# Supplementary figures and images for: Worldwide suicide mortality trends by firearm (1990–2019): A joinpoint regression analysis
Source: PLoS One. 2022 May 25;17(5):e0267817. doi: 10.1371/journal.pone.0267817 (PMC9132310; doi:10.1371/journal.pone.0267817)

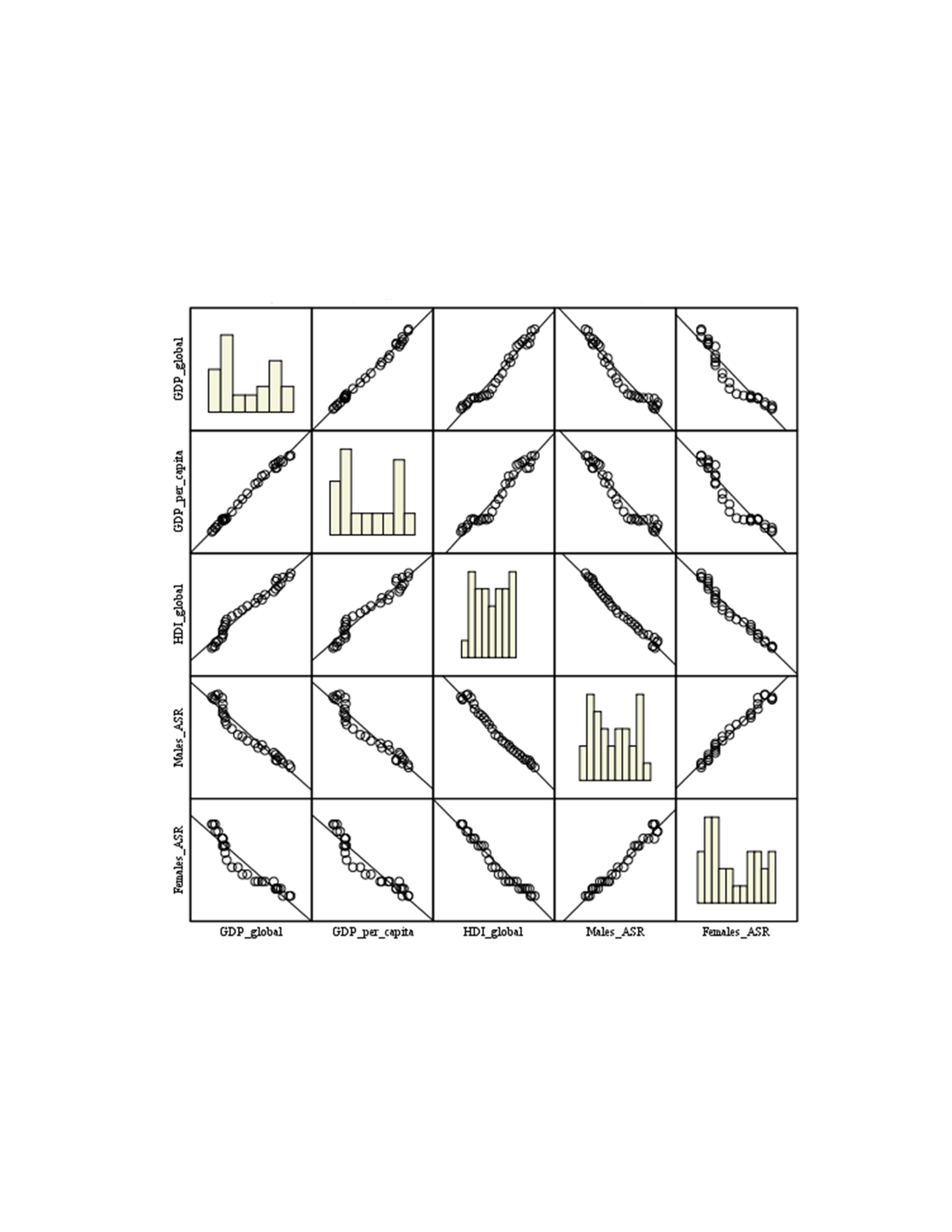

Supplement: S1 Fig — (TIF) [file pone.0267817.s001.tif]

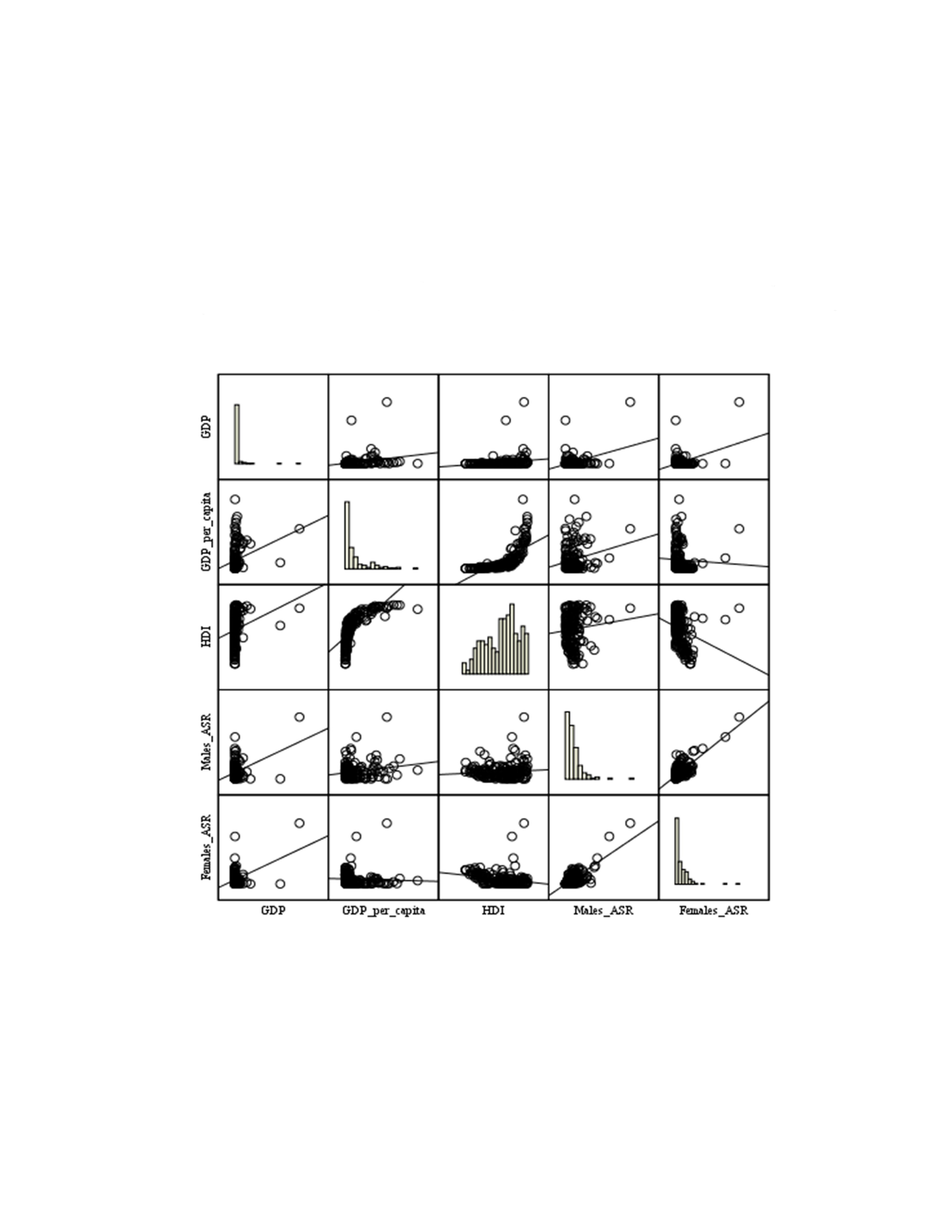

Supplement: S2 Fig — (TIF) [file pone.0267817.s002.tif]
