# Supplementary material for: Worldwide suicide mortality trends by firearm (1990–2019): A joinpoint regression analysis
Source: PLoS One. 2022 May 25;17(5):e0267817. doi: 10.1371/journal.pone.0267817 (PMC9132310; doi:10.1371/journal.pone.0267817)
Supplement: S1 Table — (DOC) [file pone.0267817.s003.doc]

| S1 Table. Mortality of suicide by firearm, by location and sex, 1990-2019; a joinpoint analysis* | | | | | | | | | | | | | | | | | | |
| --- | --- | --- | --- | --- | --- | --- | --- | --- | --- | --- | --- | --- | --- | --- | --- | --- | --- | --- |
|  | Age-standardized rates (per 100,000 population) | | | | | | | | | | | | | | | | | |
|  | | Both sexes | | | | |  | Males | |  | |  | Females | | |  | |  |
|  | | 1990 | | 2019 | | Trend** | | 1990 | 2019 | | Trend** | | 1990 | 2019 | | | Trend** | |
| Location | |  | | |  | |  |  | |  | |  |  | |  | | |  |
| Global | | 1.08 | | | 0.65 | | -2.0* | 1.97 | | 1.15 | | -2.1* | 0.29 | | 0.19 | | | -1.6* |
| WHO Regions | |  | | |  | |  |  | |  | |  |  | |  | | |  |
| African | | 0.66 | | | 0.61 | | -0.2 | 0.98 | | 1.04 | | +0.3 | 0.35 | | 0.21 | | | -1.9* |
| Americas | | 3.87 | | | 2.58 | | -1.4* | 7.15 | | 4.69 | | -1.5* | 0.99 | | 0.66 | | | -1.3* |
| South-East Asian | | 0.58 | | | 0.34 | | -2.0* | 0.79 | | 0.48 | | -1.9* | 0.35 | | 0.20 | | | -2.3* |
| European | | 1.33 | | | 0.66 | | -3.2* | 2.73 | | 1.33 | | -3.2* | 0.16 | | 0.07 | | | -3.3* |
| Eastern Mediterranean | | | 0.39 | | 0.35 | | -0.4* | 0.63 | | 0.56 | | -0.5* | 0.14 | | 0.14 | | | +0.0 |
| Western Pacific | | 0.14 | | | 0.05 | | -3.9* | 0.26 | | 0.09 | | -3.8* | 0.03 | | 0.01 | | | -4.6* |
| Afghanistan | | 0.40 | | | 0.37 | | -0.1 | 0.61 | | 0.47 | | -0.9* | 0.21 | | 0.27 | | | +1.2* |
| Albania | | 0.79 | | | 0.89 | | -0.5 | 1.40 | | 1.47 | | -0.5 | 0.19 | | 0.31 | | | -0.3 |
| Algeria | | 0.29 | | | 0.18 | | -1.6* | 0.47 | | 0.26 | | -2.1* | 0.11 | | 0.09 | | | +0.0 |
| American Samoa | | 1.05 | | | 0.60 | | -2.4* | 2.02 | | 1.20 | | -2.2* | 0.03 | | 0.01 | | | -4.0* |
| Andorra | | 0.82 | | | 0.42 | | -2.4* | 1.49 | | 0.80 | | -2.3* | 0.08 | | 0.05 | | | -1.8* |
| Angola | | 0.81 | | | 0.66 | | -0.7* | 1.28 | | 1.24 | | -0.0 | 0.33 | | 0.19 | | | -2.3* |
| Antigua | | 0.07 | | | 0.06 | | -0.7* | 0.13 | | 0.10 | | -1.2* | 0.01 | | 0.02 | | | +3.0* |
| Argentina | | 3.40 | | | 2.44 | | -2.2* | 6.01 | | 4.67 | | -1.9* | 1.21 | | 0.50 | | | -4.2* |
| Armenia | | 0.19 | | | 0.42 | | +2.6* | 0.35 | | 0.79 | | +2.6* | 0.05 | | 0.10 | | | +3.3* |
| Australia | | 2.73 | | | 0.58 | | -5.6* | 5.26 | | 1.14 | | -5.6* | 0.33 | | 0.06 | | | -5.8* |
| Austria | | 3.14 | | | 1.55 | | -2.9* | 6.53 | | 3.12 | | -3.0* | 0.32 | | 0.16 | | | -2.9* |
| Azerbaijan | | 0.10 | | | 0.09 | | -0.4 | 0.19 | | 0.16 | | -0.6 | 0.03 | | 0.02 | | | +1.2* |
| Bahamas | | 0.24 | | | 0.19 | | -0.9* | 0.49 | | 0.39 | | -0.9* | 0.01 | | 0.01 | | | -0.0 |
| Bahrain | | 0.20 | | | 0.10 | | -2.7* | 0.30 | | 0.15 | | -2.8* | 0.07 | | 0.03 | | | -2.2* |
| Bangladesh | | 0.45 | | | 0.15 | | -3.9* | 0.72 | | 0.25 | | -3.7* | 0.16 | | 0.04 | | | -4.4* |
| Barbados | | 0.28 | | | 0.20 | | -2.1* | 0.49 | | 0.34 | | -2.3* | 0.10 | | 0.07 | | | -1.3* |
| Belarus | | 0.49 | | | 0.41 | | -2.4* | 1.06 | | 0.93 | | -2.2* | 0.03 | | 0.02 | | | -2.0* |
| Belgium | | 2.55 | | | 1.10 | | -3.6* | 4.88 | | 2.13 | | -3.5* | 0.48 | | 0.16 | | | -4.6* |
| Belize | | 0.45 | | | 0.76 | | +0.7 | 0.78 | | 1.42 | | +0.8 | 0.10 | | 0.12 | | | +0.9* |
| Benin | | 0.76 | | | 0.77 | | +0.2 | 1.18 | | 1.31 | | +0.5* | 0.39 | | 0.29 | | | -1.2* |
| Bermuda | | 0.14 | | | 0.04 | | -4.2* | 0.28 | | 0.07 | | -4.6* | 0.02 | | 0.01 | | | -1.6* |
| Bhutan | | 0.36 | | | 0.17 | | -2.6* | 0.55 | | 0.27 | | -2.4* | 0.15 | | 0.07 | | | -3.1* |
| Bolivia | | 0.85 | | | 0.44 | | -2.6* | 1.55 | | 0.79 | | -2.5* | 0.22 | | 0.10 | | | -3.2* |
| Bosnia and Herzegovina | | | 0.64 | | 0.59 | | -0.4 | 1.29 | | 1.18 | | -0.4 | 0.04 | | 0.04 | | | -0.7* |
| Botswana | | 0.85 | | | 0.98 | | -0.3 | 1.57 | | 1.77 | | -0.5 | 0.27 | | 0.32 | | | +1.1* |
| Brazil | | 1.68 | | | 0.53 | | -4.4* | 3.10 | | 1.00 | | -4.3* | 0.37 | | 0.11 | | | -5.1* |
| Brunei Darussalam | | 0.07 | | | 0.05 | | -0.9* | 0.13 | | 0.08 | | -1.4* | 0.01 | | 0.01 | | | -0.0 |
| Bulgaria | | 0.65 | | | 0.76 | | -0.5 | 1.24 | | 1.42 | | -0.5 | 0.08 | | 0.12 | | | +0.3 |
| Burkina Faso | | 0.91 | | | 0.94 | | +0.2* | 1.38 | | 1.63 | | +0.7* | 0.51 | | 0.34 | | | -1.6* |
| Burundi | | 0.86 | | | 0.82 | | -0.4* | 1.40 | | 1.37 | | -0.3* | 0.41 | | 0.26 | | | -2.1* |
| CÃ´te d'Ivoire | | 1.08 | | | 1.02 | | -0.3 | 1.71 | | 1.69 | | -0.2 | 0.38 | | 0.28 | | | -0.9* |
| Cabo Verde | | 1.16 | | | 1.06 | | -0.8* | 2.42 | | 2.05 | | -1.2* | 0.21 | | 0.16 | | | -1.2* |
| Cambodia | | 0.18 | | | 0.10 | | -2.1* | 0.35 | | 0.18 | | -2.3* | 0.04 | | 0.02 | | | -3.0* |
| Cameroon | | 0.73 | | | 0.85 | | +0.6* | 1.07 | | 1.45 | | +1.2* | 0.41 | | 0.29 | | | -1.4* |
| Canada | | 3.92 | | | 1.47 | | -3.7* | 7.50 | | 2.87 | | -3.7* | 0.51 | | 0.13 | | | -5.1* |
| Central African Republic | | 1.17 | | | 1.47 | | +0.9* | 2.01 | | 2.72 | | +1.2* | 0.44 | | 0.38 | | | -0.5* |
| Chad | | 0.76 | | | 0.98 | | +1.0* | 1.11 | | 1.53 | | +1.3* | 0.44 | | 0.40 | | | -0.4* |
| Chile | | 2.42 | | | 0.57 | | -5.4* | 4.66 | | 1.09 | | -5.4* | 0.38 | | 0.09 | | | -6.0* |
| China | | 0.10 | | | 0.03 | | -4.5* | 0.17 | | 0.05 | | -4.5* | 0.02 | | 0.01 | | | -3.3* |
| Colombia | | 1.79 | | | 0.93 | | -2.9* | 3.39 | | 1.80 | | -2.8* | 0.28 | | 0.12 | | | -3.8* |
| Comoros | | 0.46 | | | 0.45 | | -0.4* | 0.60 | | 0.72 | | +0.5* | 0.32 | | 0.22 | | | -2.1* |
| Congo | | 0.91 | | | 0.64 | | -1.5* | 1.57 | | 1.07 | | -1.6* | 0.36 | | 0.24 | | | -1.6* |
| Cook Islands | | 0.86 | | | 0.46 | | -1.9* | 1.56 | | 0.94 | | -1.5* | 0.09 | | 0.03 | | | -4.0* |
| Costa Rica | | 1.43 | | | 1.16 | | -1.5* | 2.72 | | 2.29 | | -1.4* | 0.18 | | 0.14 | | | -1.1* |
| Croatia | | 2.93 | | | 1.34 | | -4.4* | 5.79 | | 2.71 | | -4.2* | 0.37 | | 0.11 | | | -6.6* |
| Cuba | | 0.64 | | | 0.20 | | -5.0* | 1.21 | | 0.34 | | -5.5* | 0.05 | | 0.06 | | | +1.0* |
| Cyprus | | 0.87 | | | 0.64 | | -1.2* | 1.73 | | 1.28 | | -1.2* | 0.03 | | 0.01 | | | -4.5* |
| Czechia | | 1.22 | | | 0.92 | | -1.2* | 2.50 | | 1.83 | | -1.2* | 0.09 | | 0.08 | | | -2.0* |
| DPR Korea | | 0.15 | | | 0.10 | | -1.5* | 0.29 | | 0.18 | | -1.7* | 0.03 | | 0.01 | | | -2.7* |
| DR Congo | | 0.63 | | | 0.75 | | +0.9* | 1.01 | | 1.34 | | +1.3* | 0.29 | | 0.24 | | | -0.8* |
| Denmark | | 2.65 | | | 0.66 | | -4.9* | 5.35 | | 1.32 | | -4.9* | 0.08 | | 0.02 | | | -4.9* |
| Djibouti | | 0.42 | | | 0.54 | | +0.9* | 0.58 | | 0.82 | | +1.3* | 0.25 | | 0.22 | | | -0.7* |
| Dominica | | 0.16 | | | 0.15 | | -0.7* | 0.31 | | 0.26 | | -0.8* | 0.03 | | 0.03 | | | -0.0 |
| Dominican Republic | | 0.74 | | | 0.89 | | +1.4* | 1.41 | | 1.65 | | +1.3* | 0.11 | | 0.13 | | | +0.5 |
| Ecuador | | 0.77 | | | 0.52 | | -2.3* | 1.41 | | 0.99 | | -2.1* | 0.16 | | 0.07 | | | -4.1* |
| Egypt | | 0.23 | | | 0.22 | | +0.5* | 0.37 | | 0.34 | | +0.2* | 0.07 | | 0.09 | | | +1.5* |
| El Salvador | | 0.42 | | | 0.15 | | -4.1* | 0.80 | | 0.30 | | -3.9* | 0.08 | | 0.03 | | | -4.1* |
| Equatorial Guinea | | 0.97 | | | 0.43 | | -3.2* | 1.74 | | 0.78 | | -3.2* | 0.36 | | 0.15 | | | -3.4* |
| Eritrea | | 0.77 | | | 0.94 | | +0.8* | 1.27 | | 1.61 | | +1.0* | 0.40 | | 0.37 | | | -0.1 |
| Estonia | | 1.98 | | | 0.71 | | -5.2* | 4.27 | | 1.49 | | -5.2* | 0.07 | | 0.02 | | | -4.8* |
| Eswatini | | 0.87 | | | 1.44 | | +2.6* | 1.61 | | 2.86 | | +2.8* | 0.30 | | 0.32 | | | +1.2* |
| Ethiopia | | 1.06 | | | 0.53 | | -2.6* | 1.41 | | 0.79 | | -2.0* | 0.69 | | 0.25 | | | -4.0* |
| Fiji | | 0.08 | | | 0.05 | | -1.7* | 0.14 | | 0.08 | | -2.1* | 0.02 | | 0.02 | | | +0.5 |
| Finland | | 8.22 | | | 2.01 | | -5.3* | 16.57 | | 3.93 | | -5.4* | 0.32 | | 0.13 | | | -3.4* |
| France | | 5.16 | | | 1.70 | | -4.2* | 10.10 | | 3.45 | | -4.1* | 0.92 | | 0.20 | | | -6.2* |
| Gabon | | 0.70 | | | 0.64 | | -0.2 | 1.23 | | 1.20 | | +0.1 | 0.25 | | 0.15 | | | -1.9* |
| Gambia | | 0.60 | | | 0.73 | | +0.7* | 0.88 | | 1.23 | | +1.2* | 0.29 | | 0.25 | | | -0.7* |
| Georgia | | 0.39 | | | 0.61 | | +1.4* | 0.78 | | 1.26 | | +1.5* | 0.07 | | 0.03 | | | -3.5* |
| Germany | | 1.27 | | | 0.60 | | -2.9* | 2.69 | | 1.20 | | -3.0* | 0.14 | | 0.05 | | | -4.2* |
| Ghana | | 0.52 | | | 0.44 | | -0.2 | 0.79 | | 0.76 | | +0.3* | 0.26 | | 0.18 | | | -1.0* |
| Greece | | 0.68 | | | 0.66 | | -0.0 | 1.31 | | 1.29 | | +0.1 | 0.10 | | 0.06 | | | -1.5* |
| Greenland | | 27.43 | | | 14.11 | | -2.4* | 46.70 | | 24.52 | | -2.3* | 5.58 | | 2.69 | | | -3.1* |
| Grenada | | 0.09 | | | 0.03 | | -3.2* | 0.16 | | 0.06 | | -3.4* | 0.03 | | 0.01 | | | -4.3* |
| Guam | | 2.34 | | | 1.46 | | -2 1* | 4.17 | | 2.80 | | -1.8* | 0.19 | | 0.02 | | | -9.2* |
| Guatemala | | 1.41 | | | 0.39 | | -5.9* | 2.70 | | 0.75 | | -6.0* | 0.18 | | 0.07 | | | -4.0* |
| Guinea | | 0.70 | | | 0.89 | | +1.3* | 0.91 | | 1.41 | | +2.0* | 0.51 | | 0.40 | | | -0.7* |
| Guinea-Bissau | | 1.35 | | | 1.24 | | -0.0 | 2.16 | | 2.14 | | +0.3* | 0.62 | | 0.45 | | | -1.1* |
| Guyana | | 0.45 | | | 0.39 | | -0.1 | 0.85 | | 0.76 | | +0.0 | 0.07 | | 0.03 | | | -2.3* |
| Haiti | | 1.00 | | | 0.55 | | -1.9* | 1.69 | | 0.96 | | -1.9* | 0.35 | | 0.19 | | | -1.9* |
| Honduras | | 1.20 | | | 0.49 | | -3.2* | 2.25 | | 0.96 | | -3.1* | 0.20 | | 0.07 | | | -3.9* |
| Hungary | | 0.68 | | | 0.35 | | -2.9* | 1.37 | | 0.72 | | -2.7^ | 0.04 | | 0.02 | | | -3.6* |
| Iceland | | 2.26 | | | 0.87 | | -3.2* | 4.56 | | 1.73 | | -3.2* | 0.08 | | 0.02 | | | -5.6* |
| India | | 0.70 | | | 0.43 | | -1.8* | 0.88 | | 0.58 | | -1.4* | 0.50 | | 0.27 | | | -2.4* |
| Indonesia | | 0.10 | | | 0.06 | | -1.4* | 0.18 | | 0.11 | | -1.5* | 0.02 | | 0.01 | | | -1.6* |
| Iran | | 0.44 | | | 0.30 | | -1.1* | 0.69 | | 0.43 | | -1.4* | 0.19 | | 0.16 | | | -0.2 |
| Iraq | | 1.60 | | | 1.22 | | -1.2* | 2.57 | | 2.01 | | -1.0* | 0.62 | | 0.42 | | | -1.5* |
| Ireland | | 0.95 | | | 0.34 | | -4.5* | 1.87 | | 0.67 | | -4.4* | 0.06 | | 0.02 | | | -5.4* |
| Israel | | 1.77 | | | 1.01 | | -3.1* | 3.18 | | 1.88 | | -3.1* | 0.45 | | 0.18 | | | -3.5* |
| Italy | | 1.02 | | | 0.53 | | -2.7* | 2.05 | | 1.04 | | -2.8* | 0.13 | | 0.07 | | | -2.5* |
| Jamaica | | 0.05 | | | 0.55 | | +11.5* | 0.08 | | 0.94 | | +11.1* | 0.01 | | 0.17 | | | +13.8* |
| Japan | | 0.10 | | | 0.03 | | -4.4* | 0.18 | | 0.04 | | -5.2* | 0.01 | | 0.01 | | | -0.0 |
| Jordan | | 0.19 | | | 0.10 | | -2.7* | 0.28 | | 0.15 | | -2.4* | 0.10 | | 0.03 | | | -4.4* |
| Kazakhstan | | 0.29 | | | 0.25 | | -1.7 | 0.61 | | 0.52 | | -1.7 | 0.02 | | 0.02 | | | -0.3 |
| Kenya | | 0.42 | | | 0.60 | | +1.6* | 0.63 | | 1.07 | | +2.3* | 0.22 | | 0.18 | | | -0.8* |
| Kiribati | | 0.29 | | | 0.20 | | -1.4* | 0.53 | | 0.37 | | -1.3* | 0.07 | | 0.05 | | | -1.5* |
| Kuwait | | 0.09 | | | 0.07 | | -1.0* | 0.15 | | 0.12 | | -0.7* | 0.01 | | 0.01 | | | -1.8* |
| Kyrgyzstan | | 0.29 | | | 0.12 | | -4.5* | 0.62 | | 0.25 | | -4.6* | 0.02 | | 0.01 | | | -3.4* |
| Lao PDR | | 0.36 | | | 0.13 | | -3.5* | 0.66 | | 0.24 | | -3.6* | 0.08 | | 0.03 | | | -3.9* |
| Latvia | | 1.36 | | | 0.63 | | -4.5* | 2.95 | | 1.36 | | -4.5* | 0.06 | | 0.01 | | | -9.0* |
| Lebanon | | 0.21 | | | 0.16 | | -0.4* | 0.36 | | 0.27 | | -0.5* | 0.06 | | 0.06 | | | +0.6* |
| Lesotho | | 0.96 | | | 2.32 | | +4.4* | 1.87 | | 4.53 | | +4.3* | 0.24 | | 0.46 | | | +4.3* |
| Liberia | | 0.73 | | | 0.70 | | -0.3* | 1.03 | | 1.08 | | +0.1 | 0.43 | | 0.31 | | | -1.4* |
| Libya | | 0.21 | | | 0.22 | | +0.5* | 0.33 | | 0.33 | | +0.3* | 0.07 | | 0.09 | | | +1.9* |
| Lithuania | | 0.68 | | | 0.47 | | -2.6* | 1.44 | | 1.00 | | -2.5* | 0.03 | | 0.01 | | | -4.2* |
| Luxembourg | | 2.28 | | | 0.77 | | -4.3* | 4.49 | | 1.47 | | -4.4* | 0.36 | | 0.10 | | | -5.1* |
| Madagascar | | 0.59 | | | 0.60 | | +0.3* | 0.81 | | 0.95 | | +1.0* | 0.37 | | 0.26 | | | -1.3* |
| Malawi | | 0.71 | | | 0.83 | | +0.5* | 1.10 | | 1.60 | | +1.4* | 0.36 | | 0.18 | | | -2.8* |
| Malaysia | | 0.15 | | | 0.13 | | -0.5* | 0.28 | | 0.24 | | -0.7* | 0.02 | | 0.01 | | | -2.6* |
| Maldives | | 0.26 | | | 0.09 | | -4.0* | 0.44 | | 0.13 | | -4.4* | 0.07 | | 0.02 | | | -4.5* |
| Mali | | 0.82 | | | 0.73 | | -0.4* | 1.01 | | 1.04 | | +0.3* | 0.64 | | 0.42 | | | -1.7* |
| Malta | | 0.65 | | | 0.38 | | -2.1* | 1.27 | | 0.74 | | -2.2* | 0.13 | | 0.04 | | | -3.8* |
| Marshall Islands | | 1.89 | | | 1.16 | | -1.5* | 3.63 | | 2.20 | | -1.5* | 0.10 | | 0.06 | | | -2.0* |
| Mauritania | | 0.73 | | | 0.45 | | -1.5* | 0.95 | | 0.62 | | -1.1* | 0.51 | | 0.28 | | | -2.1* |
| Mauritius | | 0.13 | | | 0.08 | | -1.4* | 0.26 | | 0.15 | | -1.7* | 0.01 | | 0.02 | | | +2.0* |
| Mexico | | 0.95 | | | 0.72 | | -1.9* | 1.81 | | 1.40 | | -1.8* | 0.15 | | 0.09 | | | -2.6* |
| Micronesia | | 1.98 | | | 1.28 | | -1.5* | 3.76 | | 2.46 | | -1.5* | 0.12 | | 0.06 | | | -2.8* |
| Monaco | | 2.25 | | | 1.81 | | -0.6* | 3.36 | | 2.84 | | -0.4* | 1.29 | | 0.86 | | | -1.2* |
| Mongolia | | 0.68 | | | 0.60 | | -1.4* | 1.31 | | 1.21 | | -1.3* | 0.10 | | 0.04 | | | -3.6* |
| Montenegro | | 3.29 | | | 2.75 | | -0.7* | 5.97 | | 4.99 | | -0.7* | 0.79 | | 0.71 | | | -0.5 |
| Morocco | | 0.31 | | | 0.27 | | -0.3* | 0.55 | | 0.43 | | -0.8* | 0.08 | | 0.11 | | | +1.8* |
| Mozambique | | 0.63 | | | 1.05 | | +2.6* | 0.97 | | 2.02 | | +3.5* | 0.33 | | 0.26 | | | -0.6* |
| Myanmar | | 0.25 | | | 0.09 | | -3.9* | 0.48 | | 0.17 | | -3.8* | 0.04 | | 0.01 | | | -4.8* |
| Namibia | | 0.72 | | | 0.76 | | -0.0 | 1.29 | | 1.50 | | +0.3 | 0.24 | | 0.17 | | | -1.3* |
| Nauru | | 1.79 | | | 1.28 | | -1.2* | 3.36 | | 2.56 | | -1.0* | 0.10 | | 0.06 | | | -2.1* |
| Nepal | | 0.92 | | | 0.46 | | -2.3* | 1.69 | | 0.91 | | -2.0* | 0.15 | | 0.08 | | | -2.0* |
| Netherlands | | 0.28 | | | 0.20 | | -1.6* | 0.54 | | 0.38 | | -1.6* | 0.03 | | 0.03 | | | -1.5* |
| New Zealand | | 2.00 | | | 0.71 | | -4.0* | 3.87 | | 1.40 | | -4.0* | 0.23 | | 0.08 | | | -3.8* |
| Nicaragua | | 0.58 | | | 0.35 | | -2.4* | 1.13 | | 0.70 | | -2.3* | 0.08 | | 0.04 | | | -3.0* |
| Niger | | 0.80 | | | 0.78 | | -0.0 | 1.09 | | 1.20 | | +0.6* | 0.49 | | 0.39 | | | -1.2* |
| Nigeria | | 0.46 | | | 0.38 | | -0.5* | 0.63 | | 0.62 | | +0.1 | 0.27 | | 0.18 | | | -1.5* |
| Niue | | 0.97 | | | 0.49 | | -2.8* | 1.86 | | 0.94 | | -2.8* | 0.07 | | 0.03 | | | -3.2* |
| North Macedonia | | 0.71 | | | 0.73 | | -0.0 | 1.38 | | 1.38 | | -0.1 | 0.05 | | 0.09 | | | +1.6* |
| Mariana Islands | | 1.95 | | | 1.01 | | -2.4* | 3.48 | | 1.96 | | -2.3* | 0.04 | | 0.01 | | | -5.7* |
| Norway | | 4.45 | | | 1.11 | | -4.7* | 8.55 | | 2.13 | | -4.7* | 0.38 | | 0.07 | | | -5.7* |
| Oman | | 0.10 | | | 0.05 | | -2.2* | 0.16 | | 0.07 | | -2.4* | 0.02 | | 0.02 | | | -0.0 |
| Pakistan | | 0.37 | | | 0.36 | | -0.4* | 0.62 | | 0.62 | | -0.3 | 0.10 | | 0.10 | | | -0.3* |
| Palau | | 0.91 | | | 0.74 | | -0.6* | 1.72 | | 1.27 | | -0.9* | 0.06 | | 0.04 | | | -1.2* |
| Palestine | | 0.13 | | | 0.09 | | -1.5* | 0.22 | | 0.14 | | -1.7* | 0.05 | | 0.04 | | | -1.1* |
| Panama | | 0.53 | | | 0.18 | | -5.1* | 1.02 | | 0.33 | | -5.3* | 0.03 | | 0.02 | | | -1.6* |
| Papua New Guinea | | 0.29 | | | 0.21 | | -0.9* | 0.54 | | 0.40 | | -0.9* | 0.02 | | 0.01 | | | -1.9* |
| Paraguay | | 0.96 | | | 1.26 | | +0.7* | 1.52 | | 2.15 | | +1.0* | 0.42 | | 0.39 | | | -0.6* |
| Peru | | 0.21 | | | 0.12 | | -2.1* | 0.36 | | 0.21 | | -1.9* | 0.06 | | 0.03 | | | -3.4* |
| Philippines | | 0.14 | | | 0.11 | | -0.7* | 0.26 | | 0.19 | | -0.8* | 0.02 | | 0.02 | | | -0.0 |
| Poland | | 0.25 | | | 0.12 | | -3.2* | 0.50 | | 0.24 | | -3.2* | 0.02 | | 0.01 | | | -3.1* |
| Portugal | | 1.17 | | | 0.91 | | -0.5 | 2.34 | | 1.85 | | -0.4 | 0.16 | | 0.12 | | | -1.2* |
| Puerto Rico | | 1.96 | | | 0.65 | | -4.4* | 3.98 | | 1.33 | | -4.3* | 0.17 | | 0.05 | | | -4.8* |
| Qatar | | 0.10 | | | 0.05 | | -3.1* | 0.15 | | 0.06 | | -3.7* | 0.02 | | 0.02 | | | -0.0 |
| Republic of Korea | | 0.07 | | | 0.03 | | -3.5* | 0.15 | | 0.06 | | -3.8* | 0.01 | | 0.01 | | | -0.0 |
| Republic of Moldova | | 0.33 | | | 0.18 | | -2.1* | 0.66 | | 0.36 | | -2.2* | 0.02 | | 0.01 | | | -3.0* |
| Romania | | 0.10 | | | 0.05 | | -3.6* | 0.18 | | 0.09 | | -3.6* | 0.01 | | 0.01 | | | -0.0 |
| Russian Federation | | 0.72 | | | 0.60 | | -2.6* | 1.60 | | 1.27 | | -2.6* | 0.04 | | 0.03 | | | -1.9* |
| Rwanda | | 0.83 | | | 0.54 | | -2.6* | 1.30 | | 0.98 | | -2.0* | 0.44 | | 0.19 | | | -4.2* |
| Saint Kitts and Nevis | | 0.16 | | | 0.06 | | -3.1* | 0.25 | | 0.10 | | -3.2* | 0.08 | | 0.02 | | | -3.4* |
| Saint Lucia | | 0.35 | | | 0.26 | | -1.3* | 0.70 | | 0.49 | | -1.6* | 0.04 | | 0.03 | | | -0.8* |
| Saint Vincent | | 0.17 | | | 0.14 | | -1.1* | 0.34 | | 0.24 | | -1.5* | 0.02 | | 0.03 | | | +0.3 |
| Samoa | | 0.91 | | | 0.57 | | -1.7* | 1.68 | | 1.08 | | -1.6* | 0.07 | | 0.04 | | | -1.9* |
| San Marino | | 3.81 | | | 2.86 | | -1.0* | 7.78 | | 5.94 | | -1.0* | 0.15 | | 0.12 | | | -0.6* |
| Sao Tome | | 0.15 | | | 0.19 | | +0.7* | 0.24 | | 0.33 | | +1.1* | 0.07 | | 0.05 | | | -1.5* |
| Saudi Arabia | | 0.26 | | | 0.26 | | +0.8* | 0.34 | | 0.35 | | +1.1* | 0.15 | | 0.11 | | | -0.3 |
| Senegal | | 0.92 | | | 0.87 | | +0.1 | 1.38 | | 1.45 | | +0.5* | 0.47 | | 0.33 | | | -1.4* |
| Serbia | | 3.40 | | | 1.78 | | -2.6* | 6.25 | | 3.46 | | -2.3* | 0.69 | | 0.23 | | | -4.7* |
| Seychelles | | 0.20 | | | 0.14 | | -1.8* | 0.39 | | 0.27 | | -2.1* | 0.01 | | 0.01 | | | -0.0 |
| Sierra Leone | | 0.66 | | | 0.73 | | +0.7* | 0.96 | | 1.12 | | +0.9* | 0.35 | | 0.35 | | | +0.1 |
| Singapore | | 0.12 | | | 0.02 | | -6.8* | 0.23 | | 0.03 | | -7.6* | 0.02 | | 0.01 | | | -0.9* |
| Slovakia | | 1.69 | | | 0.73 | | -2.9* | 3.36 | | 1.43 | | -2.9* | 0.14 | | 0.08 | | | -1.8* |
| Slovenia | | 2.18 | | | 1.02 | | -3.0* | 4.60 | | 2.07 | | -3.2* | 0.08 | | 0.07 | | | -0.1 |
| Solomon Islands | | 2.81 | | | 2.02 | | -1.0* | 5.36 | | 4.00 | | -0.9* | 0.06 | | 0.03 | | | -1.8* |
| Somalia | | 0.81 | | | 1.06 | | +1.1* | 1.17 | | 1.81 | | +1.8* | 0.49 | | 0.43 | | | -0.4* |
| South Africa | | 0.85 | | | 0.68 | | -1.0* | 1.34 | | 1.24 | | -0.6 | 0.43 | | 0.19 | | | -2.9* |
| South Sudan | | 0.49 | | | 0.53 | | +0.4* | 0.66 | | 0.85 | | +1.0* | 0.27 | | 0.21 | | | -1.1* |
| Spain | | 0.53 | | | 0.33 | | -1.8* | 1.05 | | 0.65 | | -1.9* | 0.05 | | 0.03 | | | -1.5* |
| Sri Lanka | | 0.32 | | | 0.08 | | -5.9* | 0.57 | | 0.14 | | -6.0* | 0.07 | | 0.02 | | | -5.2* |
| Sudan | | 0.43 | | | 0.33 | | -0.7* | 0.73 | | 0.52 | | -1.0* | 0.13 | | 0.13 | | | +0.8* |
| Suriname | | 1.36 | | | 1.04 | | -1.2* | 2.51 | | 1.92 | | -1.3* | 0.24 | | 0.22 | | | -0.0 |
| Sweden | | 2.06 | | | 0.85 | | -3.1* | 4.07 | | 1.67 | | -3.1* | 0.13 | | 0.04 | | | -3.5* |
| Switzerland | | 7.06 | | | 1.69 | | -5.5* | 13.81 | | 3.30 | | -5.4* | 0.67 | | 0.16 | | | -5.9* |
| Syrian Arab Republic | | 0.21 | | | 0.12 | | -1.9* | 0.35 | | 0.21 | | -1.8* | 0.05 | | 0.04 | | | -1.3* |
| Taiwan | | 0.15 | | | 0.06 | | -2.9* | 0.27 | | 0.11 | | -3.0* | 0.02 | | 0.01 | | | -0.4 |
| Tajikistan | | 0.04 | | | 0.04 | | -0.5* | 0.07 | | 0.05 | | -1.3* | 0.02 | | 0.02 | | | +0.8 |
| Thailand | | 0.89 | | | 0.47 | | -3.8* | 1.67 | | 0.92 | | -3.6* | 0.15 | | 0.04 | | | -6.1* |
| Timor-Leste | | 0.18 | | | 0.11 | | -1.9* | 0.31 | | 0.19 | | -1.8* | 0.05 | | 0.02 | | | -3.3^ |
| Togo | | 0.73 | | | 0.84 | | +0.7* | 1.07 | | 1.51 | | +1.5* | 0.42 | | 0.29 | | | -1.3* |
| Tokelau | | 0.72 | | | 0.39 | | -2.2* | 1.39 | | 0.72 | | -2.4* | 0.11 | | 0.05 | | | -3.2* |
| Tonga | | 0.24 | | | 0.20 | | -0.4* | 0.45 | | 0.40 | | -0.2* | 0.04 | | 0.02 | | | -2.4* |
| Trinidad and Tobago | | 0.23 | | | 0.13 | | -2.5* | 0.45 | | 0.25 | | -2.6* | 0.02 | | 0.01 | | | -3.3* |
| Tunisia | | 0.16 | | | 0.15 | | -0.2* | 0.27 | | 0.25 | | -0.4* | 0.05 | | 0.06 | | | +0.9* |
| Turkey | | 1.65 | | | 0.64 | | -3.8* | 3.01 | | 1.12 | | -4.1* | 0.28 | | 0.17 | | | -1.3* |
| Turkmenistan | | 0.16 | | | 0.12 | | -2.1* | 0.30 | | 0.23 | | -2.3* | 0.03 | | 0.02 | | | -1.9* |
| Tuvalu | | 1.34 | | | 0.83 | | -1.4* | 2.80 | | 1.56 | | -1.8* | 0.13 | | 0.05 | | | -3.5* |
| Uganda | | 0.38 | | | 0.46 | | +0.1 | 0.62 | | 0.87 | | +0.7 | 0.15 | | 0.12 | | | -1.6* |
| Ukraine | | 0.73 | | | 1.02 | | -1.6* | 1.64 | | 2.14 | | -1.8* | 0.02 | | 0.03 | | | -0.0 |
| United Arab Emirates | | | 0.38 | | 0.33 | | -0.4* | 0.51 | | 0.41 | | -0.7* | 0.11 | | 0.10 | | | +0.3 |
| United Kingdom | | 0.37 | | | 0.14 | | -3.7* | 0.73 | | 0.26 | | -3.9* | 0.04 | | 0.02 | | | -2.4* |
| Tanzania | | 0.54 | | | 0.57 | | +0.3* | 0.87 | | 1.01 | | +0.6* | 0.25 | | 0.18 | | | -1.0* |
| United States of America | | 6.86 | | | 5.76 | | -0.5* | 12.49 | | 10.13 | | -0.6* | 1.88 | | 1.66 | | | -0.1 |
| US Virgin Islands | | 2.36 | | | 1.77 | | -1.4* | 4.80 | | 3.71 | | -1.4* | 0.22 | | 0.11 | | | -2.0* |
| Uruguay | | 4.47 | | | 3.80 | | -1.3* | 7.98 | | 6.87 | | -1.2* | 1.53 | | 1.30 | | | -1.2* |
| Uzbekistan | | 0.06 | | | 0.06 | | -0.9* | 0.08 | | 0.09 | | -0.9 | 0.03 | | 0.03 | | | -0.9* |
| Vanuatu | | 1.44 | | | 1.16 | | -1.1* | 2.72 | | 2.26 | | -1.0* | 0.09 | | 0.06 | | | -2.2* |
| Venezuela | | 1.74 | | | 2.45 | | +0.5* | 3.17 | | 4.59 | | +0.5 | 0.37 | | 0.46 | | | +0.4 |
| Viet Nam | | 0.21 | | | 0.14 | | -1.3* | 0.40 | | 0.26 | | -1.3* | 0.06 | | 0.04 | | | -1.6* |
| Yemen | | 0.38 | | | 0.37 | | +0.1 | 0.66 | | 0.60 | | -0.3* | 0.10 | | 0.15 | | | +1.5* |
| Zambia | | 0.68 | | | 0.80 | | +0.3 | 0.93 | | 1.40 | | +1.3* | 0.42 | | 0.24 | | | -2.5* |
| Zimbabwe | | 0.73 | | | 0.92 | | +1.1* | 1.28 | | 1.77 | | +1.3* | 0.24 | | 0.28 | | | +2.9* |

* Statistically significant trend (p<0.05); ** for full period presented AAPC (Average Annual Percent Change).
